# Translational Application of Biomedical Microrobots in Female Reproductive System

**DOI:** 10.1021/acsnanomed.5c00087

**Published:** 2026-01-08

**Authors:** Fatma M. Yurtsever, Martin Pumera

**Affiliations:** † Future Energy and Innovation Laboratory, Central European Institute of Technology, 48274 Brno University of Technology, Purkyňova 123, 61200 Brno, Czech Republic; ‡ Faculty of Electrical Engineering and Computer Science, VSB - Technical University of Ostrava, 17. listopadu 2172/15, 70800 Ostrava, Czech Republic

**Keywords:** biomedical microrobots, gynecological cancers, female reproductive system, personalized medicine, AI-assisted microrobots, fertility treatment, infectious disease, biomedical imaging

## Abstract

Recent advancements in robotics enable the rapid evolution of micro/nanorobotic technologies which accelerates developing precise, patient-tailored diagnostic and therapeutic strategies. Untethered microrobots are making strides in the biomedical field due to their ability to access hard-to-reach locations in the organs, allow adjusting dosage time, and can be combined with different treatments. Potential impact is evident in women’s health, where microrobotic systems may provide innovative solutions for gynecological cancers, fertility challenges, and bacterial infections within the female reproductive tract. In this perspective, we discuss the potential application of microrobots in the female reproductive system, actuation, fabrication methods, challenges, and future implications. Reproductive health is highly important for humanity’s future; therefore, it is crucial to prioritize cutting-edge research in reproductive wellness.

## Graphical abstract

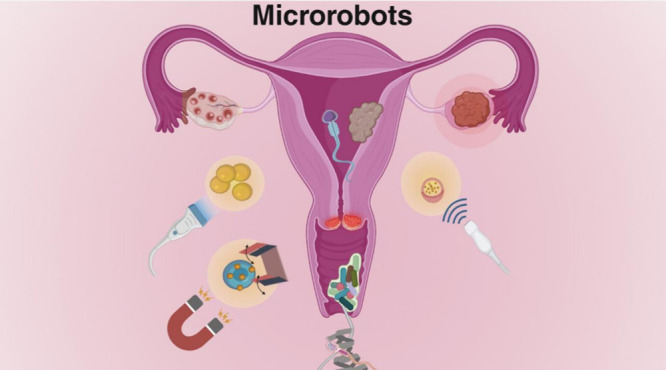

## Introduction

Micro/nanorobotics are one of the most promising alternative next-generation intelligent vesicles that can be used in various precision medicine applications, including drug, cell, and gene delivery; surgical tools for biopsy; diagnostic tools such as physical and chemical biosensors or isolation tools; biofilm eradication; and optical, ultrasonic, magnetic resonance and radionuclide imaging tools.^1–7^ The application of microrobots in reproductive medicine is one of the emerging fields to treat and diagnose infertility, cancer, infectious diseases, and other reproductive system disorders.^8–10^

The female reproductive system consists of the vagina, uterus, fallopian tubes, ovaries, and vulva, as shown in Figure 1.^11^ The majority of reported female reproductive systems diseases, such as cervical, vaginal, endometrial, and ovarian cancer, cause significant morbidity and high mortality rates.^12,13^ The most common disorders of the female reproductive system can be grouped by the primary organ affected. (i) Ovary: Key problems include ovulation-related infertility, disorders of hormone biosynthesis, ovarian cancer, polycystic ovary syndrome, and endometriosis. (ii) Fallopian tube: Ectopic pregnancy is the most frequent condition, but tubal infertility and primary cancer of the tube are also recognized. (iii) Uterine corpus: Typical issues are abnormal uterine bleeding, adenomyosis, endometrial polyps, endometritis, and endometrial cancer. (iv) Cervix: The spectrum ranges from cervical dysplasia—often a precancerous change—to invasive cervical cancer. (v) Vagina: Common disorders include pelvic organ prolapse, atrophic changes (especially postmenopause), primary vaginal cancer, and bacterial infections.^14^ It has been reported that these disorders can be treated by using drug delivery systems such as nanoparticles (NPs), intravaginal rings (IVRs), microneedle patches, vaginal films and inserts, long-acting injectables, and implants.^15^

**Figure 1:**
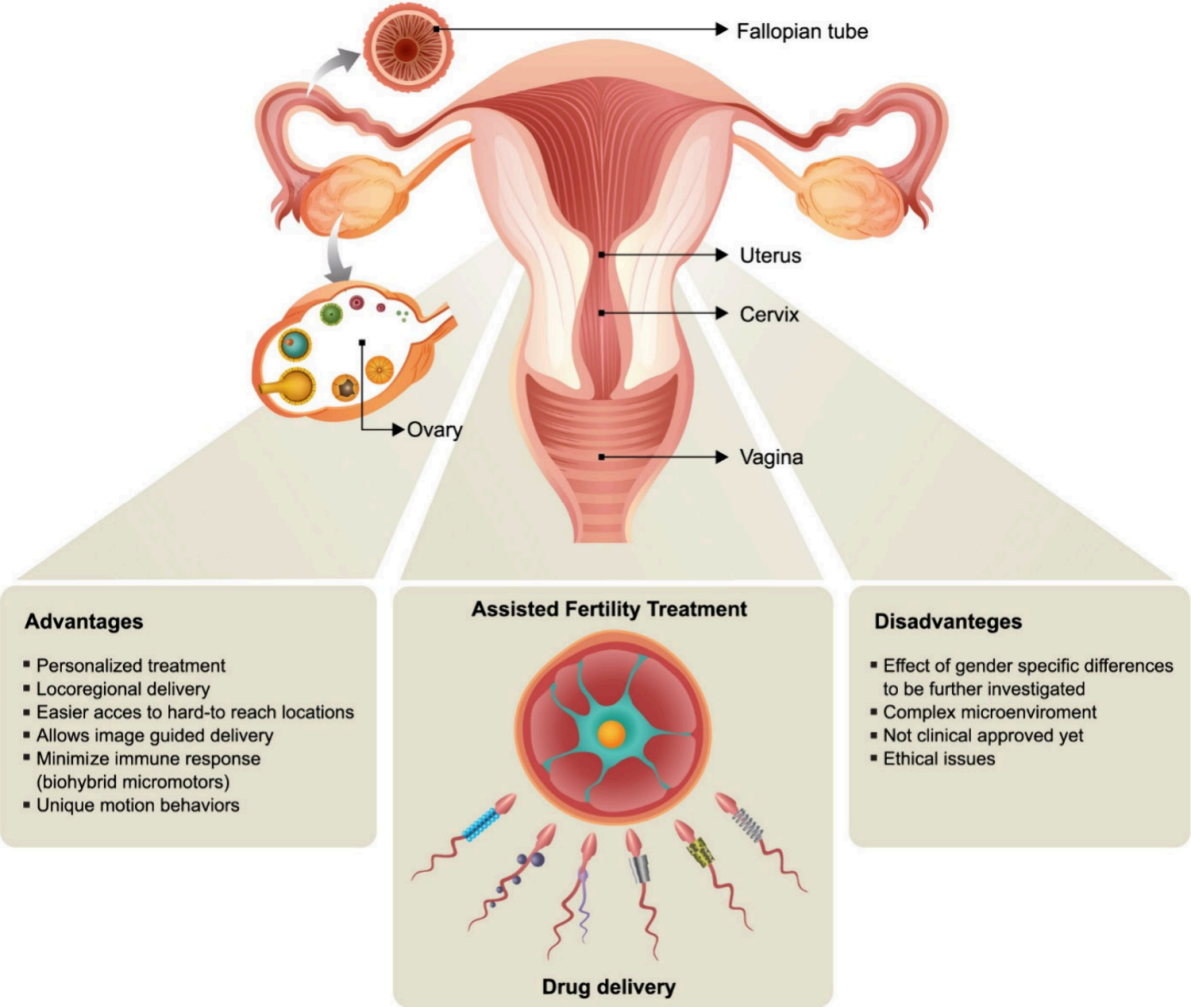
The diagram of the female reproductive system, advantages and disadvantages, and illustrations of microrobots. It has been shown in the literature that microrobots can be used for fertility treatment and drug delivery applications. It allows personalized treatment, easy access to hard-to-reach locations, and can be functionalized with imaging agents and/or therapeutics.^11,43–46^

Physiological fluids and parameters, including pH, temperature, and oxygen tension, are critical for maintaining a healthy reproductive system and equally important for developing biomedical microrobots.^16,17^ Physiological fluids such as vaginal fluid consist of epithelial cells, white blood cells, and various biomolecules, including acid phosphatase, lactic acid, citric acid, urea, vaginal peptidase, acetic acid, pyridine, squalene, and immunoglobulins.^18,19^ Moreover, the pH of each organ varies depending on the microbiome and acid–base buffering at the cellular/tissue level. For instance, the vaginal pH is around 4.42 and can reach up to the pH of 7.94 in the fallopian tubes.^18,20,21^ Low pH in the vagina is attributed to the *Lactobacillus* dominant microbiome, which generates lactic acid and hydrogen peroxide that kills the pathogen which protects the microbiome.^22,23^

### Development of Microrobots and Applications in Female Reproductive System

Microrobots are advanced intelligent microsystems that can be moved in liquid environments with different actuation modes and stimulants such as magnetic field, ultrasound, photoacoustic, or self-propulsions.^24–28^ The concept of microrobots can be linked to Richard Feynman’s lecture in 1959.^29,30^ The sperm-based biohybrid microrobot was introduced almost 50 years later as a proof-of-concept study which shows that sperms can be functionalized with magnetic nanoparticles.^31^ Afterward, microrobots have been engineered and utilized for assisted reproduction technology and anticancer drug delivery in the female reproductive system using spermatozoa as biohybrid microrobots.^32^ The sperm cells have evolved to motion devices with excellent motion properties for swimming in a complex environment, including in viscous media, acidic pH, vaginal flow back, and cervical crypts.^33–35^ Additionally, microrobots can be loaded and functionalized with bioactive molecules for therapeutic purposes.

Owing to rapid changes in lifestyle, infertility is on the rise globally, impacting 10–15% of couples worldwide.^36–38^ Low-cost, minimally invasive hormonal stimulation and intrauterine insemination, *in vitro* fertilization (IVF), or intracytoplasmic sperm injection (ICSI) are the most common procedures for infertility treatments. However, the success rate of these technologies falls between 42% and 47%.^8^ The bottleneck in IVF/ICSI treatments is associated with the female age, endometrial lining, poor oocyte activation, implantation failure, altered hormonal environment, and poor semen quality.^39,40^ The success rates of the treatments are measured by fertility rate, implantation rate, clinical pregnancy rate, and live birth rate.^41^ However, there are some limitations to overcome critical barriers for the successful translation of IVF treatment,^42^ for instance, biocompatibility and/or biodegradability, the ability to capture and secure the gametes/embryo during transport, the ability to navigate through physiological conditions, access to the secreted molecules either by the oviduct ciliary cells or embryo, dimensions must be smaller than the oviduct size (<500 μm), efficient movement in viscoelastic media and against the flow produced by peristaltic motion and cilia beating in the fallopian tube, and preserving the oviduct. Medical microrobots are one of the promising alternatives to overcome the implantation failure of current infertility treatments in reproductive medicine.^42^ Figure 1 illustrates the female reproductive system including advantages/disadvantages and application of microrobots such as microdrillers, microhelices, sperm tail decorated magnetic beads, microclaws, artificial flagella, and inorganic membranes to transport single immotile/motile sperm for assisted fertility treatment.^43–45^

Gynecological disorders, in particular, ovarian, vaginal, cervical, and endometrial cancers, show significantly high mortality and morbidity rate, which require an urgent need for alternative treatment. Gynecological cancers are currently treated with a combination of surgical, chemotherapeutic, and radiotherapeutic interventions. Developing targeted locoregional drug delivery systems that provide effective doses in a controlled way is one of the main goals in the worldwide fight against cancer.^47–49^ Current treatment methods are limited due to the complex tumor microenvironment, high metastatic spread, and developed multidrug resistance^50^ which results in high cytotoxicity and low bioavailability. Local delivery of therapeutics will eliminate the systemic circulation and hepatic clearance which will enhance the drug concentration in the tumor microenvironment. Therefore, developing biomedical microrobots are one of the most promising alternatives which can externally be controlled and deliver the cargo to targeted regions to diagnose and treat gynecological cancer.^47^ Moreover, biomedical microrobots can be tuned and bioengineered for personalized treatments to minimize immunogenicity and enhance the bioavailability of active pharmaceutical ingredients. Competent microrobots for the treatment of female reproductive system-related disease, infections, and assisted fertility treatment require the following properties including (i) biocompatibility, (ii) minimized immunogenicity, (iii) high maneuverability, and (iv) high stability in different pH, temperature, viscosity, and flow conditions. However, further studies need to be conducted to understand nonspecific attachment in tissues to complex microenvironments, to track microrobots using medical image guided techniques, and to enhance biocompatibility for further clinical studies.

### Actuation and Propulsion

The microrobots can be actuated by external force or self-propulsion while mimicking self-organization and stimuli response to the environmental changes. Although there are many different propelling mechanisms to actuate microrobots such as chemically powered, light powered, ultrasound, electric field, and photoacoustic, most of the literature related to the microrobots in the female reproductive system is focused on the most promising physical actuator, which is the magnetic field. The main limitations of photoacoustic and light-powered actuation have restricted penetration depth due to the high scattering and absorption of tissues^10^ while the magnetic field actuation showed high tissue penetration capacity and was proven safe under the magnetic field of 8T.^51,52^ The overall speed of the microrobots in the reproductive tract is lower than 1 mm/s due to the viscous environment with a required penetration depth of 50 mm.^8^

Magnetic microrobots can be attached to sperm tails for external actuation.^43^ The artificial microstructures’ and sperm swimming propulsive performances depend on their helical shape^53^ and viscosity in low Reynold numbers.^43^ The helical shape shows a linear increase in the velocity as the frequency increases. However, the motion and maneuverability still need to be improved to implement them in real conditions. Spermatozoa and similar microorganisms can move in high-viscosity mediums using cilia or rotating propellers called flagella with a corkscrew, beating, or waving motion with 10 times larger bending waves than bacteria flagella. Their motion in synthetic viscoelastic fluids showed higher propulsion in non-Newtonian fluids than Newtonian fluids of the same viscosity.^54^ Passive helical filaments of bacterial flagella are actuated by the head (body) of the rotary microrobots.

Unique navigational properties of human sperm cells make them excellent candidates to deliver anticancer drugs to hard-to-reach areas in the female reproductive system, such as ovarian cancer lesions. As illustrated in Figure 2, therapeutics like doxorubicin (DOX), incorporated into the sperm head, which were then magnetically guided, have been reported as a potential model for treating cervical and other gynecological cancers.^47,55^ Medina-Sánchez et al. reported on DOX-loaded bovine sperms by coincubation methods. Cell viability studies indicated that the tumor-killing range reached 87%, whereas the free DOX treatment group was 55%.^56,57^

**Figure 2:**
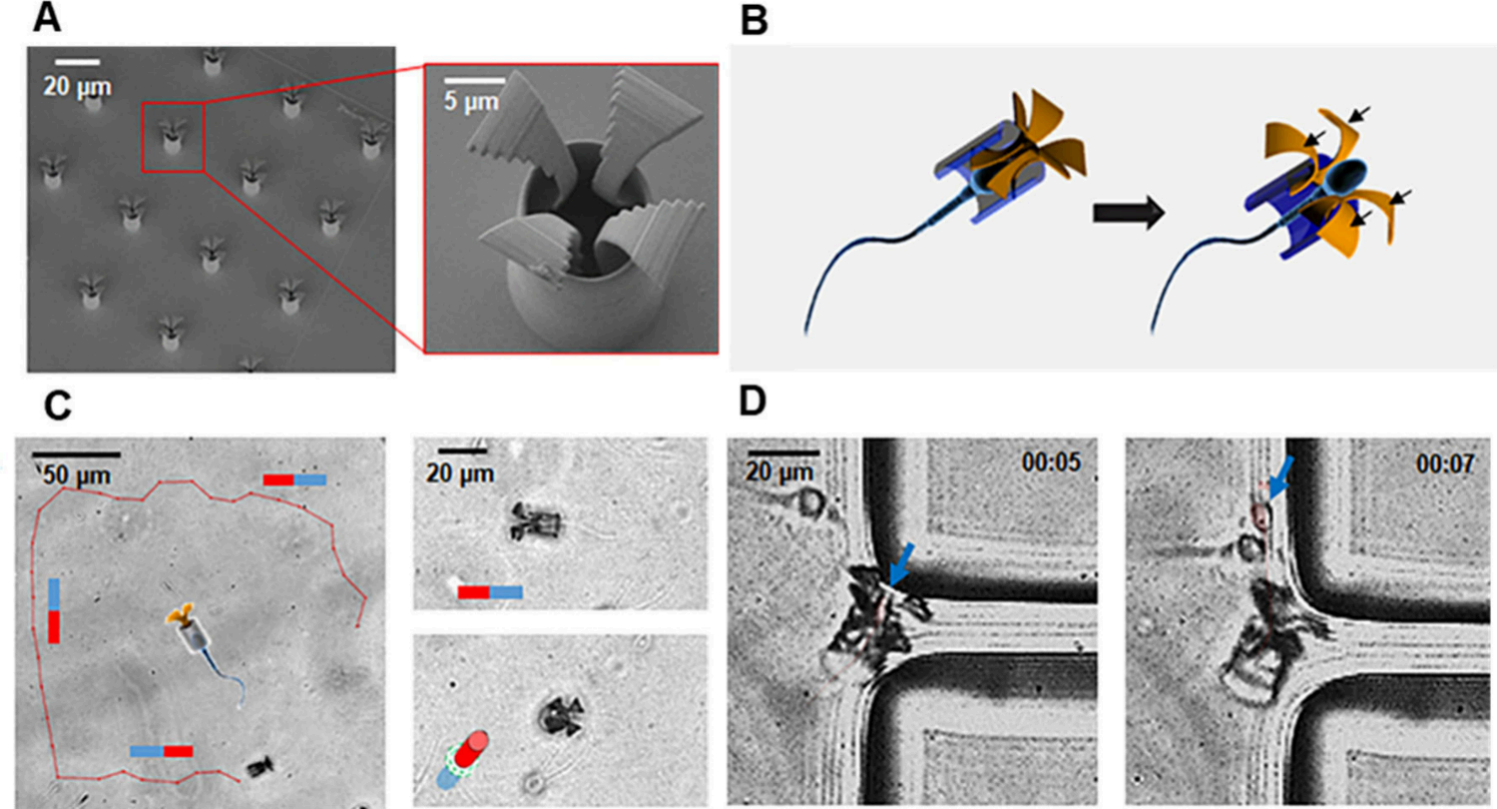
Magnetically controlled biohybrid sperm cells are possible drug carrier vehicles with high encapsulation capacity, minimized cytotoxicity, and high stability. (a) Electron microscopy images of a tetrapod structure with a tubular body and four flexible bent arms. (b) Illustration of sperm releasing from tetrapod microrobots. (c) Tracking the magnetic motion of a tetrapod microrobots. (d) Real-time imaging of sperm release when the flexible arms are in contact with the PDMS wall. Reproduced from ref 47. Copyright 2017, American Chemical Society

Schmidt et al. reported on human spermbots to treat 3D cultures of cervical and ovarian cancer with combinatorial drug therapy with doxorubicin (DOX) and camptothecin (CPT).^58^ The study demonstrated that more than 94% of cancer cells were detached after 4 days of treatment with a human spermbot loaded with DOX and CPT. It was also mentioned that the sperms not only promote navigation but also serve as an active protective layer for the anticancer drug. In summary, drug delivery application of microrobots were successfully demonstrated in various applications using biohybrid microrobots to deliver cancer therapautics.^59,60^

### Materials and Fabrication Methods

Material selection of the microrobots is highly critical to ensure biosafety and biocompatibility. These microrobots can be fully degradable or retrieved from the female reproductive system using external forces without requiring additional surgical operation. Soft scaffolds of the micromotor can be provided by using alginate, gelatin, gelatin methacrylate (GelMA), collagen, or silk while the actuation of the microrobots can be sustained by incorporating magnetic (superparamagnetic iron oxide nanoparticles, SPIONs), FePt, NdFeB, CrO_2_, and BaFe_12_O_19_), ultrasound (SiO_2_ based),^61^ or photoacoustic (Au-decorated micromotors)^62^ imaging.^8,61,63^ Microrobots for reproductive system applications can be fabricated by using different methods, including template-assisted methods, 3D/4D printing, ultraviolet (UV) polymerization, two-photon polymerization (TPP), soft lithography, stop-flow lithography (SFL), etc., in which soft and smart materials are patterned in the desired geometry.^33,64^ Incorporating soft and smart materials through a two-photon lithography process enables precise control over the microcarriers’ properties and functionalities. The TPP method enables fabricating microrobots with a uniform finish and within sub-100 nm resolution.^65^ Although the TPP method provides fabrication of featured geometries, it is expensive and can be considered a slow method for clinical applications. Moreover, the biocompatible ink options are limited for the TPP method such as acrylates, collagen, bovine serum albumin, fibrinogen, and modified ceramic and silica with different photoemitters including methylene blue, rose Bengal, eosin, and Irgacure 2959.^66^ Schmidt et al. fabricated a microcarrier via the two-photon polymerization (TPP) method to address the issues of low sperm cell count and their motility in male infertility treatment to overcome the biological barrier for oocyte fertilization.^46^ Two different inks were used including a nonstimuli-responsive polymer, specifically IPS photoresist and thermoresponsive hydrogel poly­(*N*-isopropylacrylamide) (PNIPAM). It has been demonstrated that the combination of pH and temperature-responsive material in the microcarriers effect the motility of the sperm cell.^46^ Moreover, it enhances the removal of the extracellular matrix of the cumulus cell of the oocyte which facilitates sperm–oocyte interaction. 3D/4D printing such as direct laser printing (DLP) and stereolithography (SLA) provides a broad ink type which can be printable including gelatin methacrylate-based PEGDA, composite inks with ceramics, live organisms, and cells.^67^ Therefore, it is one of the promising techniques for developing personalized microrobots for translational medicine application in the female reproductive system. However, the resolution and scaling down of the geometries are still limited.

Microrobots can be designed via different approaches, including biohybrid or bioinspired approaches. Microorganisms are one of the main inspirations for developing biocompatible self-propelled or externally propelled microrobots. Bioinspired microrobots mimic the natural motion and function of the biological organisms which are potential candidates for assisted reproductive technologies.^68^ For instance, micromotors with flexible joints imitate human hands and provide grasping behavior which is an alternative approach for single cell manipulation.^69,70^ Also, the microrobot with soft segments mimics the flagellar motion of the bacteria and spermatozoa.^71,72^ Most of the studies were focused on biohybrid microrobots in the female reproductive system (Figure 3).

**Figure 3:**
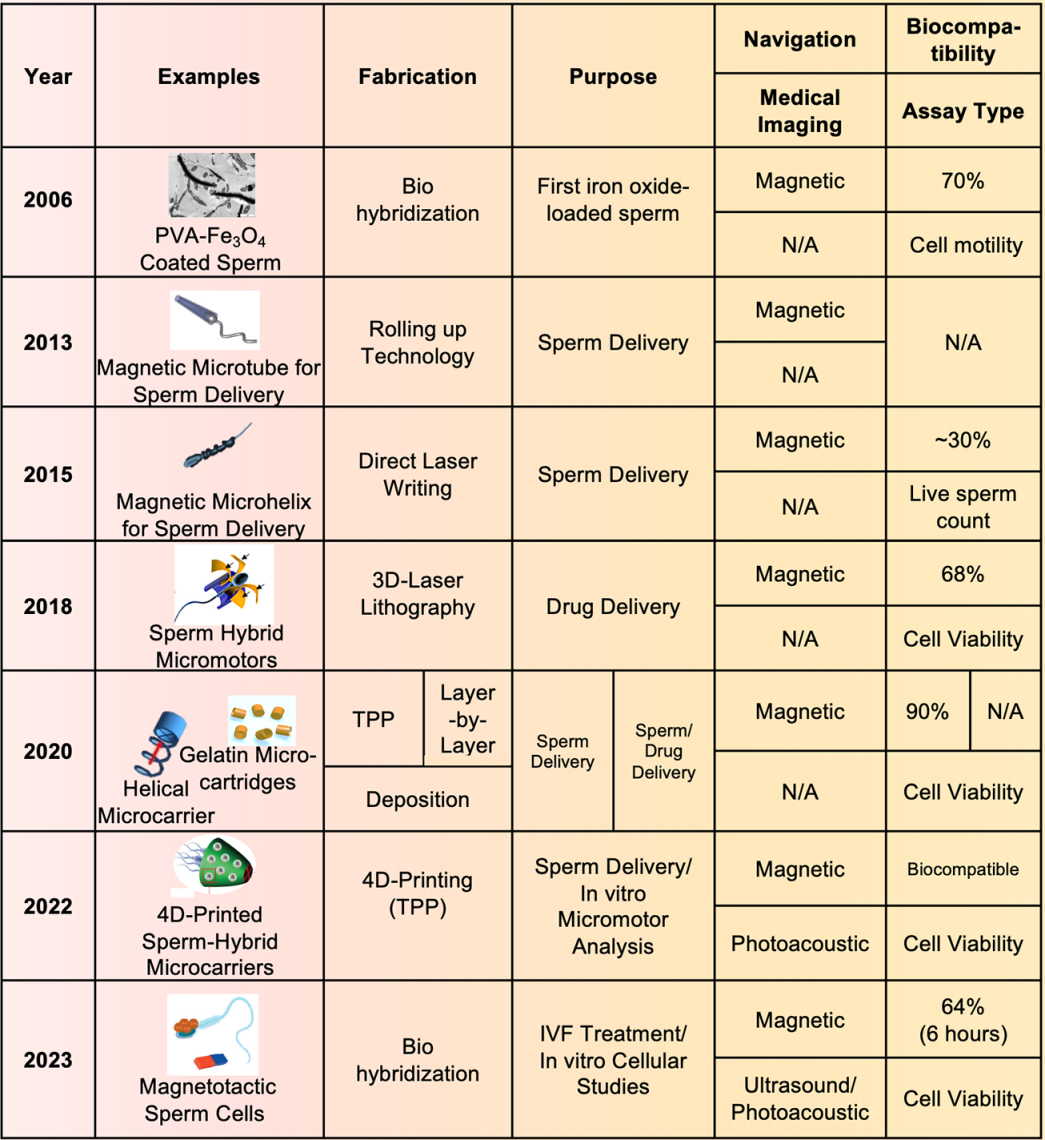
General timeline of the development of microrobots for the reproductive system. 2006—The magnetite-coated biohybrid sperm was demonstrated and its ability of motility and acrosome reaction which indicates the active fertility capacity was tested. Reproduced from ref 31. Copyright 2006, American Chemical Society. 2013—the proof of concept magnetic microtube for externally controlled motion of a single sperm was reported. Reproduced or adapted with permission from ref 87. Copyright 2013, Wiley. 2015—Artificially motorized sperm cells using magnetic microhelices for transportation of the sperm cell to the oocyte for assisted fertility treatment was developed couple of years later. These polymer-based microhelices were prepared by using a direct laser writing method and coated with Ni and Ti for magnetic navigation and to improve biocompatibility, respectively. Overall, in vitro studies showed that the application of microrobots in fertility treatment is an alternative approach to enhancing treatment efficiency. Reproduced from ref 44. Copyright 2015, American Chemical Society. 2018—a laser printed tetrapod microstructure was developed to carry drug-loaded sperms in the female reproductive tract with using a magnetic propulsion mechanism. Reproduced from ref 47. Copyright 2018, American Chemical Society. 2020—protein (bovine serum albumin, BSA)-based hyaluronic acid microflakes on magnetic helical shaped microcarriers were reported. Protein-based microflakes enhance the binding of sperm on the micromotors, while magnetic microhelices drive micromotors to the oocytes. Afterward, the protein structure was hydrolyzed by a local protease enzyme. It was also demonstrated for drug loading and carrying on sperm, which show there is no leakage nor sperm escape obtained during transportation. Reproduced from ref 88. Available under a CC-BY-NC license. Copyright 2020, Wiley-VCH Verlag GmbH & Co. KGaA. There was another study that was reported related to template-assisted gelatin-based microcartridges called spermbot for transportation of the sperm to the oocyte for assisted fertility treatment. Reproduced from ref 36. Copyright 2020, American Chemical Society. 2022—4D-printed multifunctional microcarriers were printed using two-photon-lithography techniques and coated with heparin as well to promote pH and temperature responsive release and motion performance. These microcarriers were coated with gold to photoacoustic imaging. Those microcarriers were designed and fabricated to carry around 10 sperm cells in one structure. Reproduced from ref 46. Available under a CC-BY-NC license. Copyright 2022, Wiley-VCH GmbH. 2023—researchers reported the coupling of magnetic microparticles to spermatozoa and investigated the viability, motion, and imaging using ultrasound and a photoacoustic method. Reproduced from ref 68. Available under a CC-BY-NC-ND license. Copyright, 2023, John Wiley and Sons. It was shown that incorporating the magnetic microparticles did not influence the viability of the microrobots; motility of the microrobots was enhanced twice compared to the microcarriers previously published (ref 54).

Biohybrid microrobots like spermbots provide high-motion precision, high power-to-weight ratio, and elevated flexibility and enhance the biocompatibility of microrobots. For instance, the motion behavior of the spermatozoa and flagellum-like bacteria has sufficient motion across the low Reynold’s number regime. Among bacteria and algae, sperm has additional advantages as it does not cause any immune response and has unique motion behavior regardless of fluid flow, which can move chemotaxis (chemical), thermotaxis (temperature), thigmotaxis (near-surface effects), and rheotaxis.^73–79^ Most sperm-based microrobots depend on the self-propulsive force of active flagellum and are sensitive to physiological conditions (pH, temperature, and chemicals).^75,80–83^ All of these advantages promote better navigation inside the body for targeted drug delivery. However, sperm-based biohybrid microrobots have low coupling efficiency.^84^ There are various strategies to decorate sperm of bacteria-based biohybrid microrobots with nanoparticles to enhance imaging and enable external actuation and cargo delivery. These are (i) electrostatic interactions between negatively charged sperm or bacteria membranes and positively charged nanoparticles, (ii) noncovalent bonding of nanoparticles on sperm to maintain sperm motility (for instance, biotin–streptavidin interactions are well-known noncovalent ligand protein interactions), and (iii) covalent surface functionalization such as amide, thiol–maleimide, or click-chemistry reactions.^32,43,85,86^ There were reported motorized sperm cells with magnetic microhelices to transport immotile but functional sperm to the oocyte for infertility treatment.^44^ The microcartridges were prepared with stimuli-responsive behavior in order to release the sperm by pH change via heparin coating on gelatin-based microcartridges.

Microrobot motility can be enhanced by the addition of caffeine in the medium of the sperm bots. In addition, biofunctionalization of the microtubes with fibronectin or hyaluronic acid to improve the coupling efficiency results in enhancement of spermbot performance.^89^ Magnetically driven microtubes and a synthesized protein-based hyaluronic acid (HA) microflake (MC3) are utilized to transport multiple motile sperm cells. The motion behavior was monitored in different viscosity ranges, simulating oviduct fluid, saliva, and blood.^88^ It has also been reported that the length of the microtubes affects the performance of the spermbots. As the microtubes were shorter, spermbots moved faster.^89^

### Translational Perspectives of Microrobots

Translational studies of biohybrid and bioinspired microrobots in the female reproductive system have been getting attention and are promising to treat disease and assist fertility treatment (Figure 4). Several microrobots are under evaluation for minimal to noninvasive anticancer interventions.^79^ However, to date, none have been explored to diagnose or treat issues with the female reproductive system at the clinical level. Similarly, microrobots can be a promising approach to treating infectious diseases in the female reproductive system, particularly as an effective alternative for high doses of antibiotic treatment. The microrobots can deliver antibiotic drugs locally, which might enhance biodistribution and increase treatment efficiency to tackle issues related to bacterial infections, such as bacterial vaginosis (BV).

**Figure 4:**
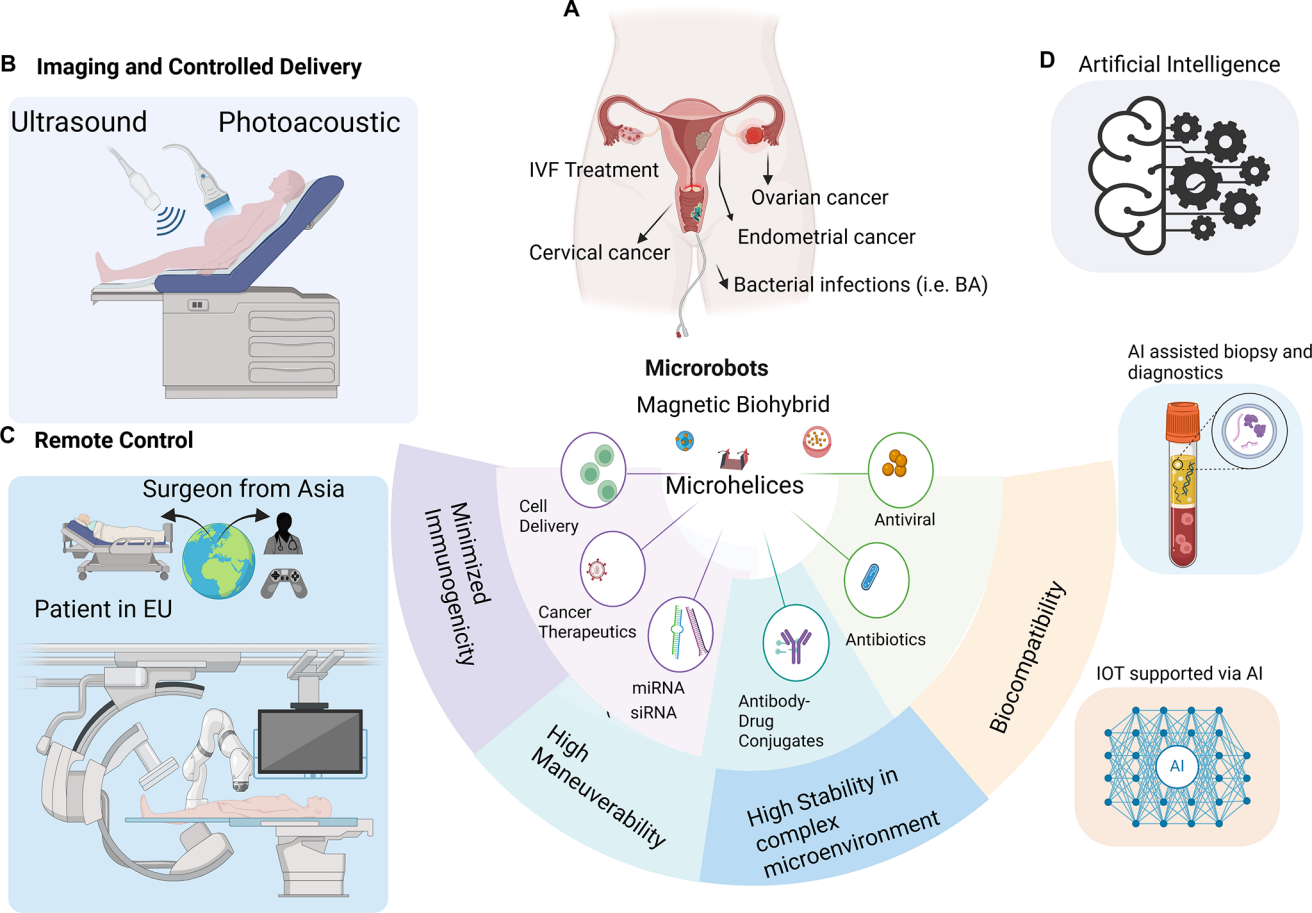
Scheme of perspective in the translational application of microrobots in precision medicine and personalized diagnostic and treatment applications. (A) Illustration of the most common female reproductive system disorders, required characteristics, possible application of microrobots, and incorporating therapeutics with microrobots. (B) Schematic of diagnostic, motion control of microrobots that can be possible with ultrasound and photoacoustic methods. (C) Microrobots can be remotely monitored with real-time imaging techniques by medical professionals. (D) Implementation of artificial intelligence technology offers a great opportunity to improve the minimal invasive diagnostics methods including biopsy and controlled motion behavior.

Characterization of endometrial microbiota and tissue sampling is critical for early diagnostics of female-specific cancers and for predicting fertility issues.^90,91^ Microrobots can also be utilized for tissue or liquid biopsy in the female reproductive system.^92^ Unlike large scale robotic biopsy tools, microrobots can move inside the body with minimal invasion and high precision.^93^ In addition, microrobots can collect liquid samples. Moreover, specific DNA mutations associated with ovarian or endometrial cancer can be detected with an on-the-fly mode microrobot sensor.^94^

The microrobots can be used in medical image-guided locoregional drug delivery with ultrasound, X-ray fluoroscopy, magnetic resonance imaging (MRI), and computer tomography (CT). Incorporating clinically used contrast agents with microrobots allows one to monitor the locomotion using X-ray fluoroscopy. This application will enhance the efficiency of cancer treatment as it allows precise control and monitoring of drug delivery in the female reproductive system for precision medicine.

Alternatively, ultrasound and photoacoustic manipulations can be feasible approachs to image and navigate the microrobots since the ultrasound transducers have already been FDA approved and already been used in clinical applications.^95^ It has been shown that ultrasound microswimmers are fabricated by a 3D-nanoprinted technique using Nanoscribe. The 3D-nanoprinted microrobotic swimmers were propelled and manipulated using a single ultrasound transducer by oscillating air bubbles inside the microrobotic swimmers’ cavity.

Although, the ultrasound-based microrobots have been reported in the literature, they have not yet been fully understood regarding their motion and real-time imaging in the female reproductive system yet. Therefore, there is still a need to discover precise control inside the physiological environment. The ultrasound-based microrobots can move autonomously by taking advantage of high energy density and low attenuation, which may help to move the hard-to-reach point in the female reproductive system. Physiochemical conditions including bubble generation, oscillation, and expansion and mechanical vibration of the fluid causes microstreaming which results in enhancing the ultrasound actuation of microrobots.^96^ Compares to ultrasound-based microrobots, magnetically controlled microrobots have main advantages, including that they are relatively easy to use and can be controlled precisely in complex physiological conditions. However, it is a costly method that requires a magnetic field generator and controller.

Artificial intelligence (AI) offers a promising solution to control and analyze the motion of the microrobots, particularly in biomedical applications such as female reproductive health.^97^ Incorporation of AI technology into microrobots can enhance their precision, autonomy, and ability to respond dynamically to complex biological environments. In female reproductive health, AI-powered microrobots have great potential to transform diagnostics, treatment, and drug delivery. For instance, microrobots could be used to navigate inside the reproductive system with high accuracy, targeting specific areas to treat conditions, such as endometriosis, uterine fibroids, or certain gynecological cancers. AI can help these microrobots analyze real-time data, adjust their movements, and optimize their therapeutic actions based on each patient’s specific needs.^98^ Additionally, AI algorithms can assist in monitoring reproductive health by analyzing patterns in data collected from microrobots. This could lead to early diagnostics and a better understanding of reproductive system disorders and personalized treatment plans. For fertility treatments, AI-enhanced microrobots might assist in more precise sperm or egg delivery, potentially improving success rates in procedures such as in vitro fertilization (IVF). Incorporating AI into microrobots offers a future where minimally invasive, precise interventions in female reproductive health become more effective, reducing side effects and enhancing patient outcomes. Implementation of artificial intelligence and deep learning-based approaches to navigate and perform the predecided tasks using predefined algorithms can be another approach to monitor and control the collective behavior of untethered microrobots in the female reproductive system.^99,100^

## Conclusion and Outlook

Microrobots can be adopted as an advanced technology for translational medicine applications, including drug delivery, IVF treatments, biopsy, minimally invasive medical imaging, and treating bacterial infections in the female reproductive system. The ability to fine-tune size, structure, and biocompatibility makes them an ideal candidate for personalized medicine applications.^101^ As advanced intelligent drug delivery platforms, the untethered microrobots minimize systemic and cytotoxicity and enhance bioavailability during locoregional administration. Moreover, recent studies show microrobots designed and engineered for X-ray, ultrasound, magnetic resonance, and acoustic imaging.^102^ However, clinical and regulatory evaluations are essential before adoption of these technologies. Achieving this will require seamless collaboration among academic, pharmaceutical, and biomedical sectors and swift translation of research into practice. Although the microrobots physical, mechanical, and in vitro drug release tests can be conducted according to the FDA device guidelines for drug eluent stents and in vitro vaginal rings, there are still regulatory approvals of the microrobots for clinical applications that remain a significant challenge due to the novelty of the materials, reproducible fabrication method, in vivo degradation, precise drug-release kinetics, actuation mechanism, real-time imaging, in vitro–in vivo correlation, large-scale manufacturing, long-term biosafety, and patient-specific variability requirements.
